# Viral filtration using carbon‐based materials

**DOI:** 10.1002/mds3.10107

**Published:** 2020-07-12

**Authors:** Rupy Kaur Matharu, Harshit Porwal, Biqiong Chen, Lena Ciric, Mohan Edirisinghe

**Affiliations:** ^1^ Department of Mechanical Engineering University College London London UK; ^2^ Department of Civil Environmental and Geomatic Engineering University College London London UK; ^3^ School of Engineering and Materials Science Queen Mary University of London London UK; ^4^ School of Mechanical and Aerospace Engineering Queen's University Belfast Belfast UK

**Keywords:** antiviral, graphene, graphene nanoplatelets, graphene oxide, nanomaterials, nanosheets

## Abstract

Viral infections alone are a significant cause of morbidity and mortality worldwide and have a detrimental impact on global healthcare and socio‐economic development. The discovery of novel antiviral treatments has gained tremendous attention and support with the rising number of viral outbreaks. In this work, carbonaceous materials, including graphene nanoplatelets and graphene oxide nanosheets, were investigated for antiviral properties. The materials were characterized using scanning electron microscopy and transmission electron microscopy. Analysis showed the materials to be two‐dimensional with lateral dimensions ranging between 1 and 4 µm for graphene oxide and 110 ± 0.11 nm for graphene nanoplatelets. Antiviral properties were assessed against a DNA virus model microorganism at concentrations of 0.5, 1.0 and 2.0 wt/v%. Both carbonaceous nanomaterials exhibited potent antiviral properties and gave rise to a viral reduction of 100% across all concentrations tested. Graphene oxide nanosheets were then incorporated into polymeric fibres, and their antiviral behaviour was examined after 3 and 24 hr. A viral reduction of 39% was observed after 24 hr of exposure. The research presented here showcases, for the first time, the antiviral potential of several carbonaceous nanomaterials, also included in a carrier polymer. These outcomes can be translated and implemented in many fields and devices to prevent viral spread and infection.

## 1 INTRODUCTION

Viruses are intracellular pathogens that pose an ever‐increasing danger to human health due to the emerging and remerging of disease. They cause infection by penetrating and reproducing inside living host cells, consequently causing cellular damage and illness. Viruses enter the body through all possible routes and spread from one person to another through direct or indirect contact with the pathogen (Baron, 1996). Viruses have been shown to survive in aerosols for up to 3 hr, on copper for 4 hr, on soft surfaces (fabric) for 12 hr and on hard non‐porous surfaces for several days (Bean et al., 1982; Doerrbecker et al., 2011; van Doremalen et al., 2020). In addition, their high viral mutation rates make treatment difficult. Though substantial effort has been devoted to producing efficacious clinical therapies for viral infections, the number of permitted antiviral medications is inadequate. A comprehensive review in 2016 identified only 90 antiviral drugs were approved for the treatment of 22 viral infections since 1963, with no treatment for over 200 infectious diseases (De Clercq & Li, 2016). Along with antiviral drugs, vaccines are efficacious in preventing and treating viral infections. However, the success of vaccines in a pandemic relies on several factors, including early detection and the availability of specific vaccines (Monto, 2006). This phenomenon constitutes a public threat, caused by the increased morbidity and mortality, additional costs due to the excessive use of antiviral medications and the subsequent burden on healthcare systems.

Efforts on the next generation of antivirals have been focused on nanomaterials, particularly metallic nanoparticles (Ganesh, Venkatakrishnan, & Tan, 2019; Kanematsu et al., 2019; Ray et al., 2018; Reddy et al., 2018). Carbon‐based nanomaterials, such as graphene and graphene oxide, hold distinctive characteristics that have attracted significant attention in a variety of applications. Graphene oxide is one of the most extensively explored materials for a wide range of applications. Traditionally, graphene oxide is formed from the chemical exfoliation of layered crystalline graphite into mono‐sheets (Boehm, Setton, & Stumpp, 1994; Park & Ruoff, 2009); however, alternative methods, such as self‐assembly (Tang et al., 2012) and chemical vapour deposition (Liu & Chen, 2016), have been proposed. Graphene oxide is a non‐stoichiometric chemical compound comprised of a single atomic plane of carbon molecules arranged in a regular hexagonal lattice with carboxylic groups at its edges and hydroxyl groups in its basal plane (Compton & Nguyen, 2010; Dideikin & Vul, 2019; Park & Ruoff, 2009). Graphene nanoplatelets are composed of layers of carbon atoms arranged in a honeycomb structure (Georgakilas, Perman, Tucek, & Zboril, 2015; Li et al., 2014). Each atom is attached to three neighbouring carbon atoms in the x‐y plane by sigma bonds (Scida, Stege, Haby, Messina, & García, 2011). The atoms also have a weakly delocalized π‐electron cloud that is orientated in the z‐axis (Greshnov, 2014; Luo, Qiu, Lu, & Ni, 2013; Novoselov et al., 2007). The antibacterial properties of carbonaceous nanomaterials have been widely investigated and reported in the literature; however, their antiviral properties are unknown (Matharu, Ciric, & Edirisinghe, 2018; Matharu, Ciric, et al., 2018; Matharu et al., 2018, 2020). Studying the interaction between graphene nanomaterials and viral pathogens is of immense interest, to allow for the development of new antiviral treatments. In particular, the use of polymer carriers and their manufacturing techniques for the incorporation of carbonaceous materials are particularly relevant because of the emergence of coronaviruses (Perlman, 2020; Wang, Horby, Hayden, & Gao, 2020).

In this research, the inhibitory effects of graphene nanoplatelets and graphene oxide nanosheets on *Escherichia coli* T4 bacteriophage (DNA virus) were investigated. The carbonaceous nanomaterials were then incorporated into polymeric fibres, and the antiviral activity of the resulting fibres (carriers) was studied to determine whether or not the materials remain effective.

## 2 MATERIALS AND METHODS

### 2.1 Materials

#### 2.1.1 Nanomaterials

Graphene oxide nanosheets were prepared from graphite powder following a modified Hummers' method (Marcano et al., 2010). Graphene oxide nanosheet synthesis and characterization has previously been reported (Matharu et al., 2020). Grade C‐750 graphene nanoplatelets (nanoplatelet size <2 µm with a typical thickness of 2 nm) were purchased from XG Sciences.

Poly(methyl methacrylate) (PMMA) (Mw 120,000 g/mol) and chloroform were acquired from Sigma‐Aldrich and used during fibre manufacture. All solvents and chemicals were of analytical grade and used as received.

#### 2.1.2 Microbial strains and media

*Escherichia coli* T4 bacteriophage (ATCC 11303‐TB4) was used as the model organism throughout these experiments. Freeze‐dried cultures were sourced from LGC Standards and cultured following manufacturers’ instructions. Stock cultures of *E. coli* ATCC 11303 were stored in a Microbank^TM^ at −20°C, while the bacteriophage was kept at 2°C. Antiviral activity was assessed against this microorganism as it is commonly available and safe to work with in Biosafety Level 2 laboratories.

ATCC Medium 129 was prepared using sodium chloride, nutrient agar and nutrient broth sourced from Sigma‐Aldrich. Phosphate buffer saline was used in the antiviral studies and purchased from Sigma‐Aldrich.

### 2.2 Methods

#### 2.2.1 Nanomaterial characterization

Graphene oxide nanosheets were analysed using scanning electron microscopy (SEM, JEOL JSM‐6301F), while graphene nanoplatelets were analysed using transmission electron microscopy (TEM, JEOL JSM‐2010). Graphene oxide nanosheets were sputter‐coated with gold (Q150R ES, Quorum Technologies) for 180 s prior to imaging, while graphene nanoplatelet suspensions were prepared and drop‐casted onto carbon grids before imaging. Average diameters were estimated by measuring the width of approximately 100 specimens using ImageJ software (National Institutes of Health, Bethesda, Maryland, U.S.).

#### 2.2.2 Nanomaterial antiviral activity

T4 bacteriophage suspensions containing 0.5, 1.0 and 2.0 w/v% of graphene nanoplatelets or graphene oxide nanosheets in PBS were incubated for up to 24 hr. An actively growing broth culture of *E. coli* was prepared by incubating a single colony in sterile medium, and a plaque assay was performed to determine the virus concentration at 0, 3 and 24 hr.

#### 2.2.3 Fibre manufacture and antiviral activity

PMMA fibres containing 2 wt% of either graphene nanoplatelets or graphene oxide nanosheets were manufactured using pressurized gyration as previously reported (Matharu, Ciric, et al., 2018; Matharu, Porwal, et al., 2018; Matharu et al., 2020). The antiviral activity of the prepared fibres was assessed by incubating 0.1 g of fibres in 10 ml of T4 bacteriophage suspension for 24 hr. The number of virions present at 0 and 24 hr was counted using a plaque assay, and viral reduction was calculated. Virgin PMMA fibres were used as a control. Antiviral studies were completed in triplicate.

## 3 RESULTS AND DISCUSSION

### 3.1 Nanomaterial antiviral activity

**FIGURE 1:**
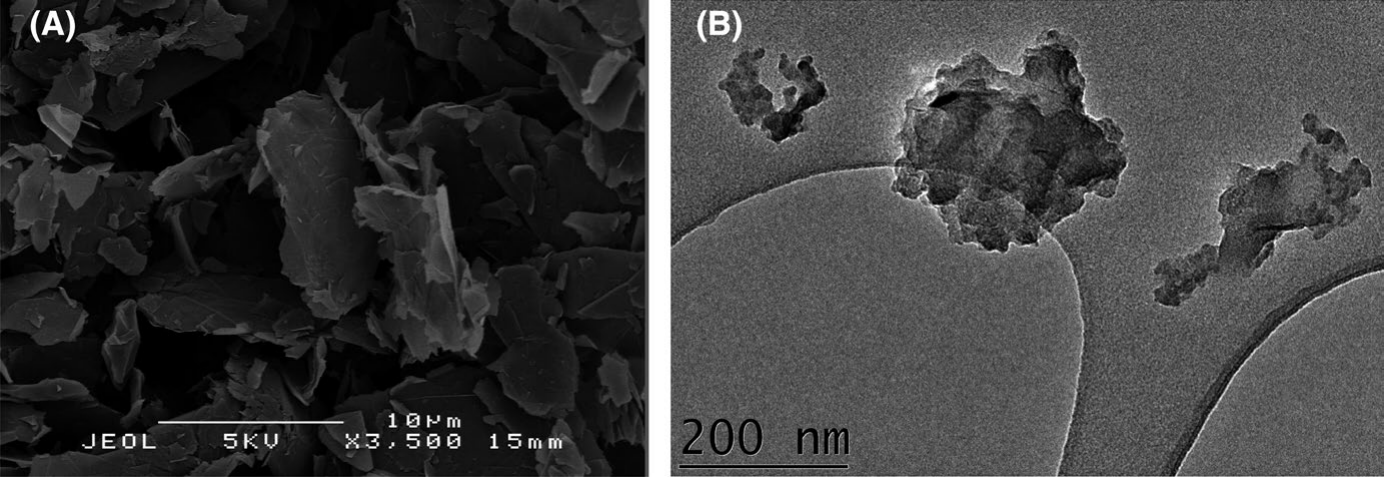
(a) Scanning electron micrograph of graphene oxide nanosheets and (b) transmission electron micrograph of graphene nanoplatelets

This research reports the effect graphene‐based nanomaterials, graphene nanoplatelets and graphene oxide nanosheets (Figure 1), have on the inhibition of viruses. Analysis showed the graphene oxide nanosheets had a thickness of 0.85 ± 0.12 nm and lateral dimensions in the range of 1–4 µm (Matharu et al., 2020), while the graphene nanoplatelets had an average width of 110 ± 0.11 nm and average length of 170 ± 0.08 nm (Matharu, Ciric, et al., 2018; Matharu, Porwal, et al., 2018). This study involves testing both nanomaterials, incorporating them into polymeric fibres and testing the resulting antiviral efficacy. Both materials were exposed to the virus for up to 24 hr, and viral reduction was calculated using a plaque assay.

**FIGURE 2:**
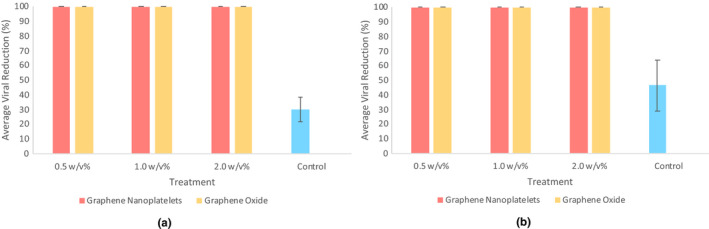
Antiviral activity of graphene nanoplatelets and graphene oxide nanosheets at 0.5, 1.0 and 2.0 w/v% against T4 bacteriophage after (a) 3 hr and (b) 24 hr of exposure. Control samples were exposed to no treatments. Error bars represent standard deviation (*n* = 3)

Graphene nanoplatelets and graphene oxide nanosheets significantly reduced virion numbers and were able to inhibit infection by 100% after just 3 hr of exposure (Figure 2). The potent antiviral activity of both graphene‐based nanomaterials is attributed to (a) firstly, its unique morphological structure and/or (b) secondly, oxidative stress caused by the production of reactive oxygen species (Krishnamoorthy, Umasuthan, Mohan, Lee, & Kim, 2012; Ye et al., 2015). A key feature of viral inactivation by carbonaceous materials is that it occurs prior to entry to the host cell (Ye et al., 2015).

Graphene‐based nanomaterials prevent viral infection by direct virion interaction caused by electrostatic and hydrophobic interactions (Donskyi et al., 2019; Ye et al., 2015). Studies have shown the sheet‐like structure of these nanomaterials to destroy the viral envelope (Sametband, Kalt, Gedanken, & Sarid, 2014; Ye et al., 2015). This subsequently prevents viral attachment and entry into the host cell, thus preventing the release of the capsid and its content. It is therefore plausible that graphene‐based nanomaterials directly interact with the virions and demolish their structure, resulting in the disruption of viral function.

Another mechanism in which graphene‐based nanomaterials cause viral inactivation is through reactive oxygen species‐related damage (Paiva & Bozza, 2014). Research has shown the inactivation of DNA viruses when exposed to hydrogen peroxide (a form of reactive oxygen species) (Hayashi, Hooper, Okuno, Takada, & Hooks, 2012). Typically, viruses produce catalase (a protective enzyme that catalyses the decomposition of oxidative stress) to prevent damage caused by reactive oxygen species. However, it is thought that most DNA viruses do not have catalase, therefore offering no protection against reactive oxygen species‐induced stress (Newcomb & Brown, 2012).

When compared to existing literature, the nanomaterials in this study had stronger antiviral properties. Ye et al. (2015) showed graphite had no antiviral properties and graphene oxide showed some antiviral activity, whereas in this study both graphene nanoplatelets and graphene oxide nanosheets showed potent antiviral activity with 100% viral reductions. In addition, when compared to conventional metallic nanoparticles, the carbonaceous nanomaterials in this study had far superior antiviral properties. Fujimori et al. (2012) reported a 50% reduction when influenza A (RNA virus) was exposed to 17 µg/ml of copper iodide nanoparticles, while other studies have reported a 50% reduction when hepatitis B (DNA virus) and human immunodeficiency virus‐1 (RNA virus) were exposed to silver nanoparticles (Fujimori et al., 2012; Lara, Ayala‐Nuñez, Ixtepan‐Turrent, & Rodriguez‐Padilla, 2010; Lu et al., 2008).

### 3.2 Fibre antiviral activity

Graphene nanoplatelets and graphene oxide nanosheets were incorporated into PMMA fibres as previously described in our antibacterial studies (Matharu, Ciric, et al., 2018; Matharu, Porwal, et al., 2018; Matharu et al., 2020), to determine whether or not they remain effective. Several methodologies have been proposed to prepare non‐leaching antimicrobial materials, but in most cases, they require a multistep procedure and are only specific for certain chemistries. Pressurized gyration approaches have demonstrated the successful manufacture of nanocomposite materials in a single step (Matharu, Charani, Ciric, Illangakoon, & Edirisinghe, 2018; Matharu, Ciric, et al., 2018; Matharu, Ciric, et al., 2018; Matharu, Porwal, et al., 2018; Matharu et al., 2020). The resulting fibre morphologies are described in Table 1.

**TABLE 1:** Fibre morphologies of the graphene oxide and graphene nanoplatelet loaded fibres produced using pressurized gyration

Nanomaterial loading (wt%)	Fibre diameter (µm)	Key microstructural features
Graphene oxide nanosheets		
0	0.75 ± 0.35	Fibres were continuous and highly porous.
2	1.44 ± 0.9	Fibres remained tubular and porous.
4	1.55 ± 0.9	Beaded fibres with a lower porosity.
8	1.99 ± 1.3	Thicker fibres with irregular particles.
Graphene nanoplatelets		
0	0.75 ± 0.35	Fibres were continuous and highly porous.
2	0.95 ± 0.40	Fibres remained tubular and porous.
4	0.99 ± 0.56	A rise in bead frequency was observed.
8	2.71 ± 1.74	Fibres were wide and heavily beaded.

**FIGURE 3:**
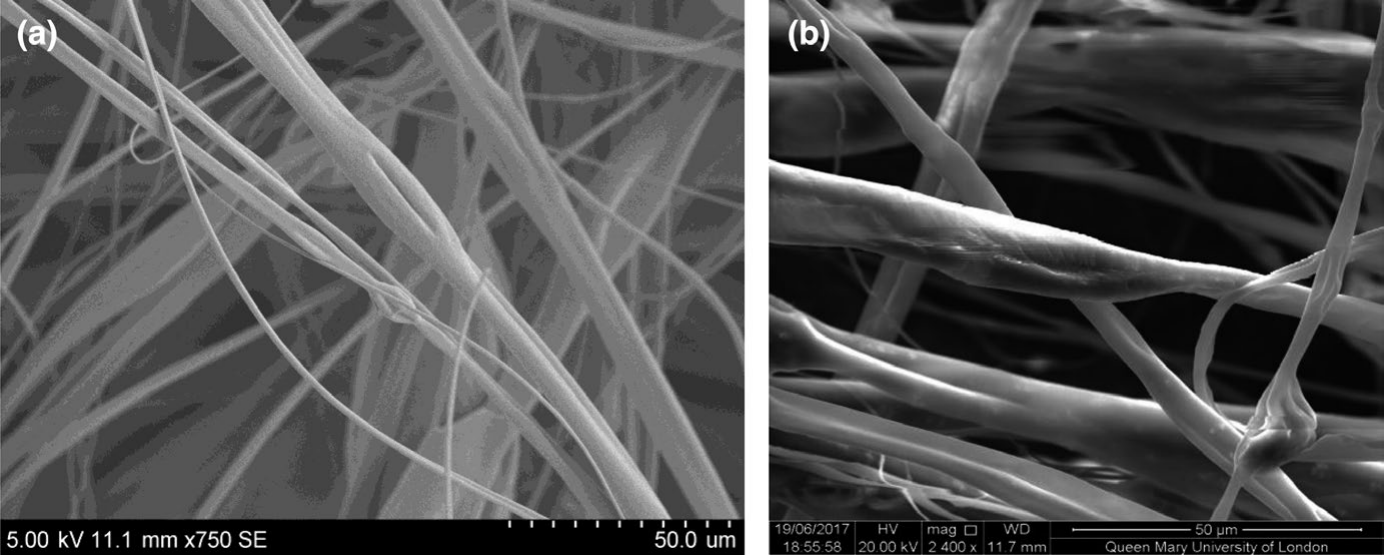
Scanning electron micrographs of poly (methyl methacrylate) fibres loaded with 8 wt % of (a) graphene oxide nanosheets and (b) graphene nanoplatelets

Figure 3 shows SEM images of the 8 wt % loaded fibres, where a smooth topography can be observed (Matharu, Ciric, et al., 2018; Matharu, Porwal, et al., 2018; Matharu et al., 2020). As none of the nanosheets are protruding from the fibre surface, the antiviral activity of the fibres is thought to be chemical rather than physical. These physical and chemical scenarios are further discussed in our antibacterial work (Matharu, Ciric, et al., 2018; Matharu, Porwal, et al., 2018; Matharu et al., 2020).

**FIGURE 4:**
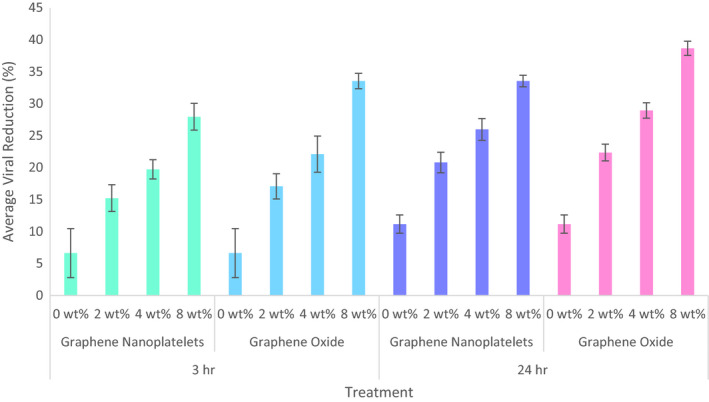
Graph showing the antiviral activity of PMMA fibres loaded with 0, 2, 4 or 8 wt % of graphene nanoplatelets or graphene oxide nanosheets against T4 bacteriophage for 3 and 24 hr. Error bars represent standard deviation (*n* = 3)

As shown in Figure 4, pure PMMA fibres (loaded with 0 wt %) showed a moderate decrease in virions. This minor reduction is likely owed to two factors: (a) the lack of host cells in the PBS to allow for viral survival and reproduction and (b) viral destruction triggered by the hydrophobic interaction between PMMA and the proteins present on the virus surface (Karlin & Brendel, 1988; Park et al., 2009; Shi & Tarabara, 2018).

Fibres containing graphene nanoplatelets and graphene oxide nanosheets exhibited antiviral properties across all conditions tested. Only a slight increase in viral reduction can be observed between the 3‐hr and 24‐hr incubation periods. This suggests a 3‐hr exposure time is sufficient for viral deactivation. After 24 hr of exposure, fibres containing the lowest nanoparticle concentration (2 wt%) exhibited viral reductions of 20.8 ± 1.6% and 22.4 ± 1.3% for graphene nanoplatelets and graphene oxide, respectively. Further increasing the loading to 4 wt % of graphene nanoplatelets or graphene oxide nanosheets resulted in viral reductions of 26.0 ± 1.7% and 28.9 ± 1.2%, respectively. Fibres containing 8 wt % graphene nanoplatelets or graphene oxide nanosheets displayed the strongest potencies with average viral reductions of 33.6 ± 0.9% and 38.7 ± 1.1%, respectively. These results indicate that the antiviral activity of the nanocomposite fibres is in fact concentration‐dependent. Increasing the loading increases the concentration of graphene‐based nanomaterials on the surface of the fibres. Therefore, more of the active agent is exposed for the virions to interact with. A study by Mori et al (2013) showed a 45% titre after exposure to silver nanoparticle/chitosan composites (embedded with 5 wt % silver nanoparticles). This result is comparable to the results obtained in this study.

Though the antiviral properties of the graphene loaded fibres were less effective when compared to pure nanoparticles, incorporating them into a polymer broadens their applications and demonstrates how they can be translated into different applications. As the graphene nanomaterials are entrapped in the fibres and not free floating, viral deactivation through direct interaction is not plausible. Therefore, only the secondary mechanism of action, production of reactive oxygen species, is functioning (Matharu et al., 2020). The unfavourable ramifications of viral pathogens are widespread, with major implications on societies, economies and political systems, especially in developing countries, not only because of the associated morbidity and mortality, but also the high cost that represents their prevention and treatment. The cytotoxicity of carbonaceous nanomaterials remains underdetermined, with existing literature giving contradictory judgements. Some studies have shown graphene nanomaterials to be toxic to mammalian cells, while other research has shown composite graphene nanomaterials to be non‐cytotoxic and can be used in a range of biomedical constructs (Gonçalves, Cruz, Ramalho, Grácio, & Marques, 2012; Liao, Lin, Macosko, & Haynes, 2011; Liu, Cui, & Losic, 2013; Pahlevanzadeh, Bakhsheshi‐Rad, & Hamzah, 2018). As the prepared composites are not intended for consumption or direct implantation into humans, toxicity is not a major concern. This study shows these nanocomposites can be used in a variety of applications, such as surface treatments, filters and more, as effective antiviral treatments.

## 4 CONCLUSIONS

In the present study, graphene nanoplatelets and graphene oxide nanosheets exhibited antiviral activity towards DNA viruses after 3 hr of exposure. The antiviral mechanism of both materials is attributed to physical and chemical interactions, with direct nanoparticle interaction being the predominant mode of action. Both graphene nanoplatelets and graphene oxide nanosheets showed comparable antiviral activity, suggesting the oxygen functional group is expendable in the antiviral mode of action.

The graphene nanoplatelet and graphene oxide polymer fibres also showed antiviral activity. The fibres show much weaker antiviral activity than the pure nanomaterials, suggesting that direct physical interaction is important for antiviral activity.

This study reports, for the first time, the antiviral activity of graphene nanoplatelets, graphene oxide and nanosheets and their resulting nanocomposite fibres with a proposal for a potential mechanism of action. As a result, nanocomposites with potent antiviral properties at low concentrations can be produced by exploiting multiple driving forces at nano‐biointerfaces due to functionalization. The data presented here shed light on antiviral development, thus helping push the industry further and alleviating the burden on global health.

## CONFLICT OF INTEREST

The authors declare no conflicts of interest. The funders had no role in the design of the study; collection, analyses or interpretation of data; manuscript writing; or decision to publish the results.

## AUTHOR CONTRIBUTIONS

R.K.M. conceptualized the study, contributed to methodology, involved in validation, performed formal analysis, involved in investigation, wrote the original draft, reviewed and edited the manuscript, visualized the data and involved in project administration. H.P. involved in investigation, provided resources, wrote the original draft, and reviewed and edited the manuscript. B.C. provided resources, wrote the original draft, and reviewed and edited the manuscript. L.C. provided resources, wrote the original draft, reviewed and edited the manuscript, and acquired funding. M.E. provided resources, wrote the original draft, reviewed and edited the manuscript, supervised the study, and acquired funding.
